# Split Pool Ligation-based Single-cell Transcriptome sequencing (SPLiT-seq) data processing pipeline comparison

**DOI:** 10.1186/s12864-024-10285-3

**Published:** 2024-04-12

**Authors:** Lucas Kuijpers, Bastian Hornung, Mirjam C. G. N. van den Hout - van Vroonhoven, Wilfred F. J. van IJcken, Frank Grosveld, Eskeatnaf Mulugeta

**Affiliations:** 1 Department of Cell Biology, Erasmus University Medical Center Rotterdam (Erasmus MC), Wytemaweg 80, Rotterdam, 3015CN The Netherlands; 2 Center for Biomics, Erasmus University Medical Center Rotterdam (Erasmus MC), Rotterdam, The Netherlands

**Keywords:** SPLiT-seq, Split-pool barcoding, Combinatorial barcoding, Data-preprocessing, Single cell RNA sequencing

## Abstract

**Background:**

Single-cell sequencing techniques are revolutionizing every field of biology by providing the ability to measure the abundance of biological molecules at a single-cell resolution. Although single-cell sequencing approaches have been developed for several molecular modalities, single-cell transcriptome sequencing is the most prevalent and widely applied technique. SPLiT-seq (split-pool ligation-based transcriptome sequencing) is one of these single-cell transcriptome techniques that applies a unique combinatorial-barcoding approach by splitting and pooling cells into multi-well plates containing barcodes. This unique approach required the development of dedicated computational tools to preprocess the data and extract the count matrices. Here we compare eight bioinformatic pipelines (alevin-fry splitp, LR-splitpipe, SCSit, splitpipe, splitpipeline, SPLiTseq-demultiplex, STARsolo and zUMI) that have been developed to process SPLiT-seq data. We provide an overview of the tools, their computational performance, functionality and impact on downstream processing of the single-cell data, which vary greatly depending on the tool used.

**Results:**

We show that STARsolo, splitpipe and alevin-fry splitp can all handle large amount of data within reasonable time. In contrast, the other five pipelines are slow when handling large datasets. When using smaller dataset, cell barcode results are similar with the exception of SPLiTseq-demultiplex and splitpipeline. LR-splitpipe that is originally designed for processing long-read sequencing data is the slowest of all pipelines. Alevin-fry produced different down-stream results that are difficult to interpret. STARsolo functions nearly identical to splitpipe and produce results that are highly similar to each other. However, STARsolo lacks the function to collapse random hexamer reads for which some additional coding is required.

**Conclusion:**

Our comprehensive comparative analysis aids users in selecting the most suitable analysis tool for efficient SPLiT-seq data processing, while also detailing the specific prerequisites for each of these pipelines. From the available pipelines, we recommend splitpipe or STARSolo for SPLiT-seq data analysis.

**Supplementary Information:**

The online version contains supplementary material available at 10.1186/s12864-024-10285-3.

## Background

The ability to analyze the transcriptome at single-cell resolution has become an increasingly important technique to uncover cellular heterogeneity within tissues, observe specific cell states, unravel gene regulatory networks and understand dynamic processes in much more detail.

**Fig. 1:**
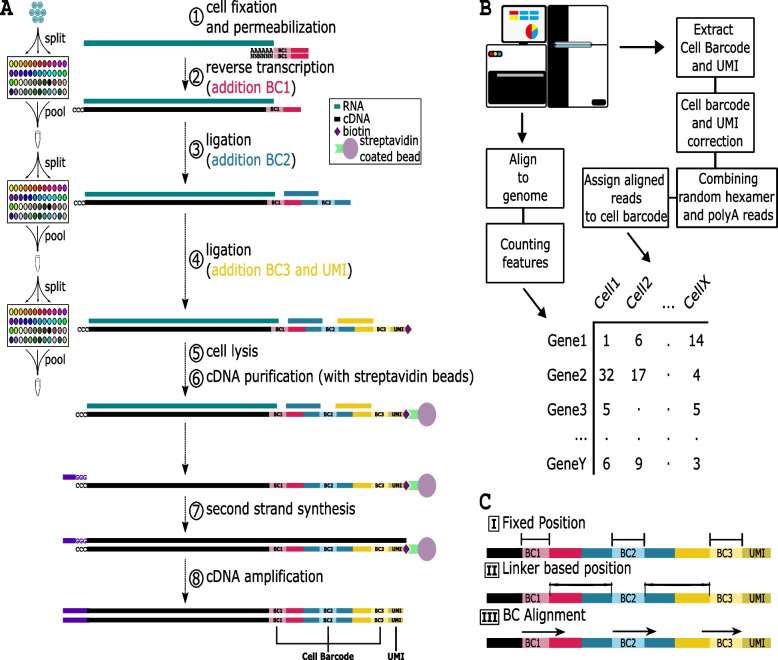
**A** Graphical description of the SPLiT-seq protocol. 1) cell fixation and ligation and permeabilization, 2) reverse transcription and addition of BarCode 1 (BC1), 3) pooling, splitting and ligation of BC2, 4) pooling, splitting and ligation of BC3 and UMI, 5) cell lysis and 6) cDNA purification, 7) second strand synthesis, 8) cDNA amplification **B**. Graphic description of the common processing steps applied by SPLiT-seq preprocessing pipelines. Depending on the pipeline steps are performed in different order or not at all (Fig S1). **C**. Depiction of the three types of computational algorithms. I) fixed position, II) linker-based position, III) BC alignment

Since its development nearly a decade ago, the single-cell sequencing field has seen a rapid growth, expanding the repertoire of improved techniques and approaches [1]. In the past years, several such technologies have been developed that allow the sequencing of the genome, epigenome, and transcriptome [2–7]. Single-cell sequencing platforms continually improve by increasing the number of cells that can be sequenced and the number of reads per cell that can be captured and importantly decreasing the cost of the experiments [6, 8, 9]. As it was the first single cell sequencing modality to arise, single-cell transcriptome sequencing has seen the most significant improvements and applications [1, 10–12]. Single-cell transcriptome sequencing techniques can be categorized into four groups: i) Droplet based approaches (e.g. 10 × genomics, Drop-seq) [13, 14], in which cells are captured in micro droplets where they are barcoded and lysed; ii) Plate based methods (e.g. SMART-seq) [15], where single cells are sorted into microwells and subsequently lysed and sequenced; iii) emulsification/gelification based techniques [16, 17] that utilize the physical separation of cells and beads within an oil or gel solution; iv) Split barcoding techniques (e.g. SPLiT-seq, sci-RNA-seq) [18, 19], in which cells are continually redistributed and new barcodes are introduced in several rounds to ensure a unique barcode combination for each cell (Fig. 1A).

Split barcoding (combinatorial barcoding) holds great potential, as it tackles two important bottlenecks in the single-cell sequencing field: cell throughput and experimental costs [18]. Albeit, split barcoding has some technical downsides and caveats: (I) An increased possibility of cell doublets due to cell clumping. (II) Not all cell types or samples can be mixed due to unequal representation of RNA molecules between samples or cell types. (III) Moreover, sample quality has to be similar to prevent sample bias, as dying cells or free-floating RNA molecules will increase background signal and mask true reads. The power of split barcoding lies in its simplicity. After fixation and permeabilization the cells go through a series of pooling and splitting. After each splitting of the cells a unique barcode is introduced which is ligated to the mRNA products (Fig. 1A). With sufficient rounds of barcoding the probability that of any two cell barcodes are the same approaches zero. The maximum available amount of unique cell barcodes can be expressed as *Number of barcodes per round*^*Rounds of split−pooling*^. This concept has no theoretical limits, but has a few practical ones. With increasing rounds there is an increasing chance of cell loss as well as aberrant ligation errors. Finally, the read length would increase with each added barcode, resulting in increased costs as well as increase sequencing error within the cell barcode. However, Split barcoding does not require any custom or specific machines nor high expertise making it accessible to every wet-lab. Split barcoding reduces the cost of single-cell sequencing experiments especially when used on a large scale [18]. Recently, SPLiT-seq has been commercialized (https://www.parsebiosciences.com) making the technique easily accessible to everyone.

Despite these advantages, the subsequent analysis of SPLiT-seq data is more complicated. Each transcript of a cell is identified through a Cell Barcode (CB), which consists of the cumulative combination of ligated barcodes and a Unique Molecular Identifier (UMI). Droplet or other micro-fluidics sequencing methods often use one barcode which is synthesized directly onto the Unique Molecular Identifier (UMI) [20, 21], while SMART-seq physically separates the cells and attaches unique sequencing adapters [20–23]. These approaches make sample deconvolution and other downstream analysis relatively easier when compared to SPLiT-seq, which uses three independent barcodes, separated by linkers (Fig. 1A), which are ligated to each other in series. Several algorithms have been generated to deconvolute the CBs and although all of them follow similar steps (Fig. 1B), they have unique approaches in how they process the reads (Fig S1). The post-sequencing algorithms can be categorized into three categories based on how they extract CBs from the read (Fig. 1C). These are: (I) Fixed position; The algorithm relies on the known theoretical BC positions relative to the start of the read. By using these positions, the algorithm extracts the CB and UMI (Table 1) [24–26] (STARsolo, splitpipe, splitpipeline, zUMI, alevin-fry splitp). (II). Linker-based position; this strategy first aligns the known linker sequences to the read, using the best alignment as a reference for the position of the barcodes and UMI (Table 1) [27, 28] (SCSit, LR-splitpipe). (III). BC Alignment; All possible expected sequences of the barcode are used to align to the read [29]. Because BCs are sampled from the same set of sequences, part of the sequence of the adjoining linker is used to anchor the barcode position (Table 1) [29] (Splitseq-demultiplex).

**Table 1:** Characteristics of the pipelines

**Pipeline**	**Compressed input**	**Linker chemistry**	**Bc sets**	**Adjustable alignment**	**Ranhex conversion**	**Algorithm**	**Programmi**n**g language**
**alevin-fry splitp** [24, 30–32]	Yes	v1,v2	Input	Yes	Yes	Position based	Rust
**LR-splitpipe** [25, 28]	No	v2	Fixed	Yes	No	Linker based position	Python
**SCSit** [26, 33]	No	v1	Fixed	Yes	No	Linker based position	C
**splitseq-demultiplex** [27]	No	Any	Input	Yes	Yes	Alignment/Position based	Python,shell
**splitpipe** [34]	Yes	v1,v2	Fixed	No	Yes	Position based	Python,shell
**splitpipeline** [16, 24]	Yes	v1,v2	Input	No	Yes	Position based	Python,shell
**STARsolo** [22, 29, 35]	Both	Any	Input	Yes	No	Position based	C,C + +
**zUMI** [23, 36]	Yes	Any	Input	Yes	Yes	Position based	Perl,R,shell,Python

Two other features of SPLiT-seq protocols are its use of different linker chemistries and random hexamers. Since its first publication [18] SPLiT-seq has been in continued development which has resulted in two different linker chemistries (v1,v2). As a feature the SPLiT-seq allows to capture as many RNA reads by using random hexamer primers in addition to Poly Adenylated (PolyA) primers. In the first barcoding round these primers are combined in the same well. Instead of using 96 barcodes only 48 are used, with the second 48 as random hexamer primers this causes that a set of barcodes originate from random hexamer primers and another from PolyA primers which are coupled through their BC sequence and well position.

In this study we compared the ease of use, performance and quality of the results generated by eight bioinformatic pipelines (alevin-fry splitp, LR-splitpipe, SCSit, splitpipe, splitpipeline, SPLiTseq-demultiplex, STARsolo and zUMI) that have been developed to process SPLiT-seq data. We provide details on their processing steps the pros and cons of these tools. Our analysis shows the importance of the chosen algorithm for the processing step, which significantly affects the downstream results and further analysis. Our findings will help others that are performing or planning to analyzing SPLIT-seq data.

## Results

### Functional comparison of data-processing pipelines

Several SPLiT-seq analysis pipelines have been developed. However, these tools have, to the best of our knowledge, not been compared and benchmarked yet in the context of SPLiT-seq. A comparison aids researchers in making an informed decision before analyzing SPLiT-seq data.

A total of 8 pipelines were found that could analyze SPLiT-seq data: alevin-fry splitp, LR-splitpipe, SCSit, splitpipe, splitpipeline, Splitseq-demultiplex, STARsolo and zUMI (Table 1). To test these pipelines, we used two published datasets: i) a small dataset [18] which contains approximately 100 mouse brain cells with 80 million reads using v1 chemistry, and ii) a larger dataset originating from PBMCs [35], containing about 15,000 cells and 2 billion reads using v2 chemistry.

We started our comparison by examining each pipeline's compatibility with input data type, algorithm utilization, the programming language, linker chemistry version, barcode sets and random hexamer conversion ability employed for each pipeline (Table 1). With regard to Input data type, out of the 8 pipelines compared, 5 can handle compressed data, optimizing disk space utilization. When comparing barcode extraction methods per algorithm, we found that the majority of pipelines employ a position-based algorithm (split-pipeline, STARsolo, zUMI, split-pipe, and alevin-fry splitp), while others like LR-splitpipe and SCSit utilize a linker-based positioning approach, and splitseq-demultiplex opts for an alignment-based positioning algorithm. These pipelines are primarily developed in Python or a combination of Python with shell scripting (used by zUMI, LR-splitpipe, split-pipeline, splitseq-demultiplex, and split-pipe). STARsolo utilizes C and C +  + , while SCSit is implemented in the C programming language and alevin-fry splitp is developed using Rust.

When we assessed the linker chemistry compatibility, except for the SCSit and LR-splitpipe pipelines, all pipelines were found capable to handle data generated using both the v1 and v2 chemistry SPLiT-seq protocols. The LR-splitpipe is originally designed to process long read sequencing data with v2 chemistry from the Oxford nanopore. Due to the technique reads are sequenced in both orientations which is considered in the LR-splitpipe algorithm. However, LR-splitpipe is relatively easily modifiable for other purposes. The barcode sequences that are used can differ in SPLiT-seq experiments. SCSit, splitpipeline and splitpipe have fixed barcode sequences within their algorithm whereas other pipelines require the sequences per position as user input. Splitpipe, however, contains all the latest barcodes published by Parse Biosciences within the program and suggests the most likely barcode set upon running the pipeline. Depending on the experiment the alignment that has to be performed might have to be altered. Splitpipe and splitpipeline currently do not contain any option to alter the alignment, in contrast to all other pipelines where the alignment is adjustable. Another important feature considered by certain pipelines is random hexamer collapsing, which sums reads from random hexamer and polyA capture to the correct CB; however, only 5 out of the 8 pipelines incorporate this collapsing step (split-pipeline, zUMI, split-pipe, alevin-fry splitp, splitseq-demultiplex). If a SPLiT-seq experiment is performed according to standard protocol algorithms such as splitpipe and splitpipeline are easy to use and everything required is already present within the program. When alterations are required for the analysis due to specific experimental or preprocessing needs other programs provide more freedom in options.

### Performance comparison

**Fig. 2:**
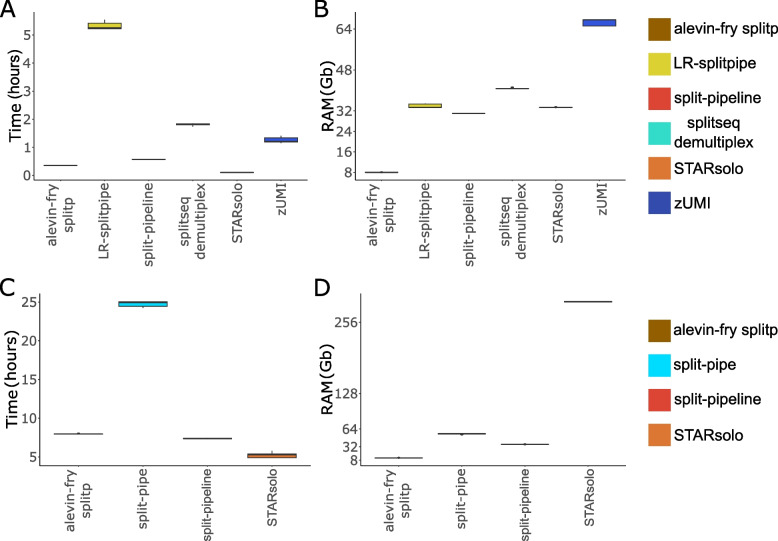
**A** Time elapsed and **B**. maximum RAM usage for each pipeline with the small dataset of 80 million reads. **C** Time elapsed and D. maximum RAM usage for each pipeline with the large dataset 2 billion reads. Each pipeline was run five times to obtain averages

Next, we compared the performance of these pipelines by first using the aforementioned small dataset containing 100 mouse brain cells. We recorded the analysis time and the memory resources needed (RAM) to process the data. The alevin-fry splitp, STARsolo and splitpipeline are the fastest pipelines when analyzing the small dataset. STARsolo completes the whole pipeline in just over 6 min (Fig. 2A). LR-splitpipe however, takes more than 5 h to complete (Fig. 2A). Besides python not being as fast as the coding language C or C +  + [34, 36] LR-splitpipe performs several steps with ‘for’ loops that parse over all the data, which are known to be slow in python [30]. RAM usage is similar for most of the compared pipelines when applied on the small dataset and range between 30 and 40 Gb (Fig. 2B). zUMI consumes a considerable higher amount of memory, while Alevin-fry only requires 10 Gb of RAM making it the most memory efficient pipeline. The SCSit pipeline was tested, but did not function properly. The pipeline crashes with an unknown error, which could not be resolved. Thus, it was not used for the rest of the study and the authors were notified. The splitpipe algorithm, published by Parse Biosciences, did not contain the right predetermined settings to run the small dataset and could not therefore not be applied. This was primarily because, the small dataset was generated using an older protocol of SPLiT-seq, which uses outdated barcoding sequences.

When testing all 8 pipelines on the larger PBMC (peripheral blood mononuclear cells) dataset zUMI, LR-splitpipe and splitseq-demultiplex required more than 5 days to finish. Therefore, those pipelines were excluded from further comparison since we deemed the long running times prohibitive for routine analysis.

When applied on the large PBMC dataset both alevin-fry and STARsolo were very fast, finishing in approximately 8 and 5 h respectively (Fig. 2C). Split-pipe took 24 h to complete. Split-pipeline, the predecessor of split-pipe, performed much better with an average run time of 7 h (Fig. 2C). When looking at memory usage alevin-fry requires minimal resources using a maximum 12 Gb of RAM (Fig. 2D). In contrast STARsolo requires 293 Gb when running the large dataset (Fig. 2D), which currently exceeds the specifications of many commercially available standard servers. Split-pipe and split-pipeline require 55 and 35 Gb of RAM respectively making it easier to run on smaller machines (Fig. 2D).

### Pipeline comparison using small mouse brain dataset

**Fig. 3:**
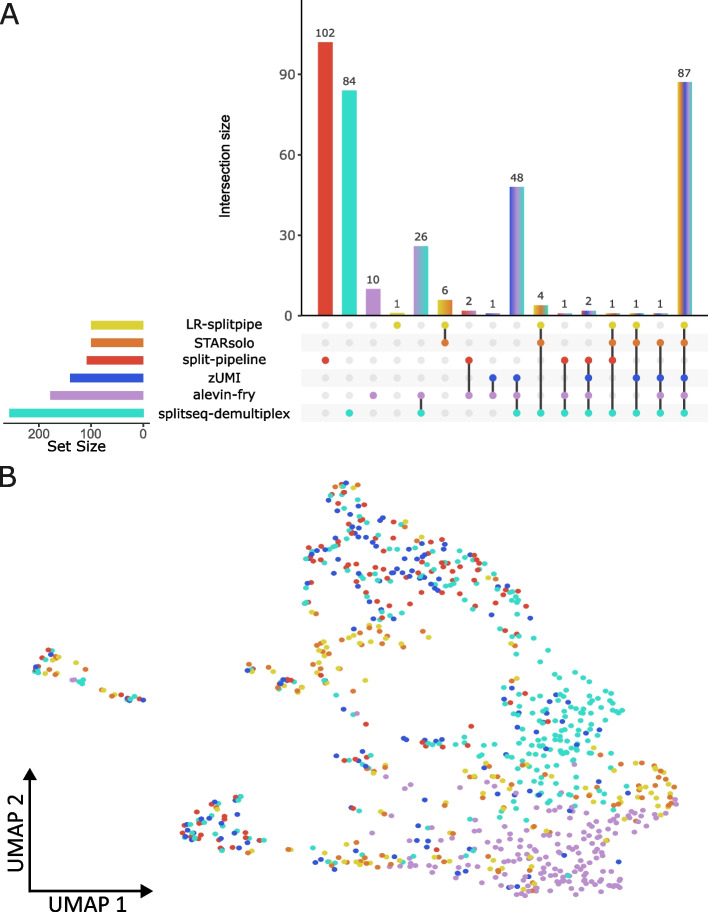
**A** An Upset plot of the cell barcodes (CB) that were generated by each pipeline after QC. Set size equals the total amount of BC generated by the pipeline. Intersection size representing the total amount of barcodes that overlap between the intersected set of pipelines. The bottom right panel shows which pipelines are being intersected. **B** A UMAP of the merged data generated by each pipeline comparing cell expression content. Cells are color-coded according to the pipeline color used in Fig. 3 A

In order to compare the pipelines in a more quantitative measure we performed further downstream analysis on the outputs of these pipelines. We first investigated the small dataset that is reported to have 100 cells that are deeply sequenced to 80 million reads [18]. Following quality control (Fig S2), the LR-splitpipe and STARsolo generated exactly 100 cells, as initially reported, while split-pipeline generated a comparable number of cells (108), which falls well within the expected range of cell counts (Table 2). zUMI’s and alevin-fry produced 140 and 178 cells respectively (Table 2). Splitseq-demultiplex finds a total of 255 cells, reaching the highest number of cells from all the compared pipelines (Table 2). When comparing the cell barcodes generated between the different pipelines 87 cells intersect between five out of the six pipelines. Surprisingly, none of those 87 cells are detected by split-pipeline (Fig. 3A). Whereas another 48 and 26 intersect between zUMI, alevin-fry and splitseq-demultiplex and alevin-fry and splitseq-demultiplex respectively, arising from their increased total cell barcodes generated (Fig. 3A). Only split-pipeline stands out having 102 unique cell barcodes that do not intersect with any cell barcodes from other pipelines. We investigated whether this was due to any technical errors such as wrong annotation of BC order e.g. BC1-BC2-BC3 vs BC3-BC2-BC1. We were unable to find the source of this phenomenon.

**Table 2:** Quantitative measurements per pipeline for the small dataset

Pipeline	Mean gene per cell	Median gene per cell	Mean umi per cell	Median umi per cell	N cells
alevin-fry	2249.9	1659	8645.5	4031.9	178
LR-splitpipe	1772.5	1179.5	4177.9	1849.5	100
split-pipeline	3602.7	2815.5	16159.1	8307.5	108
splitseq-demultiplex	1730.8	647	97746.3	9387	255
STARsolo	1755.8	1171	4045.3	1778	100
zUMI	2723.1	2233	8741.6	5137	140

When comparing gene and UMI per cell content STARsolo and LRsplitpipe perform very similar having the lowest content per cell (Table 2). Although zUMI and alevin-fry have an increased number of cells generated, they also have a higher gene and UMI content per cell, which shows differences in their counting and/or alignment compared to LR-splitpipe and STARsolo. Splitpipeline obtains the highest number of genes per cell whereas splitseq-demultiplex obtains the least number of genes per cell, despite of having the highest UMI per cell content.

To further understand and evaluate the output of these pipelines, we plotted the cells generated from each pipeline using UMAP (Uniform Manifold Approximation and Projection). This indicated that, despite their differences in quantity of content or BC tag, these cells exhibited overlapping characteristics (Fig. 3B), implying the presence of similar expression profiles. A portion of the cells produced by the alevin-fry splitp and splitseq-demultiplex pipeline tend to aggregate more closely together than cells produced by other pipelines (Fig. 3B). The aggregation of splitseq-demultiplex can be explained by the fact that it generates approximately 100 more CB than others, therefore splitseq-demultiplex is more represented within the UMAP compared to other pipelines. Alevin-fry splitp in contrast to all other pipelines uses pseudo-alignment which might result in the different expression patterns and therefore aggregation of the cells within the UMAP.

### Pipeline comparison on large PBMC dataset

**Fig. 4:**
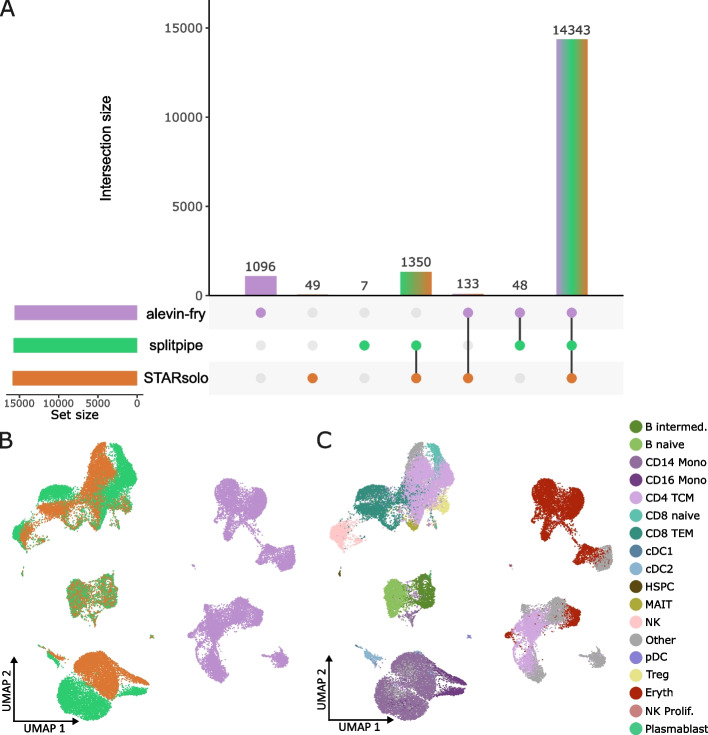
**A** An Upset plot of the cell barcodes that were generated by each pipeline after QC. Set size equals the total amount of BC generated by the pipeline. Intersection size representing the total amount of barcodes that overlap between the intersected set of pipelines. The bottom right panel shows which pipelines are being intersected. **B** UMAP after merging the data from the different pipelines together. Comparing cell expression content. Cells are color-coded according to the pipeline color used in Fig. 4**A**. **C** UMAP after merging the data from the different pipelines together. Cells are color coded by cell-type (also indicated at the right of Fig. 4**C**) after using the Azimuth cell annotation algorithm

**Fig. 5:**
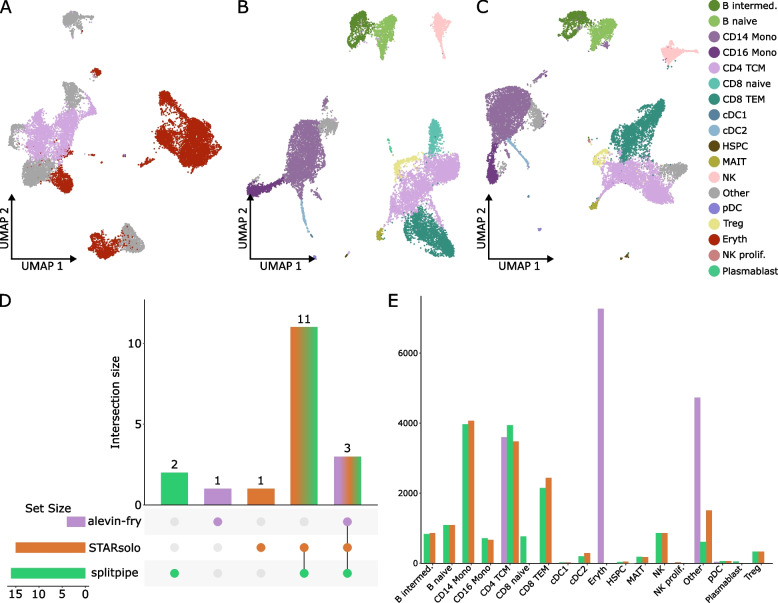
UMAP of data generated by each pipeline colored by predicted cell-type after using the Azimuth cell annotation algorithm. **A** Cell-type annotation for cells generated by alevin-fry **B** Cell-type annotation for cells generated by splitpipe **C**. Cell-type annotation for cells generated by STARsolo. Cells are color coded (also indicated at the right of Fig. 5**C**) by cell-type after using the Azimuth cell annotation algorithm. **D** An Upset plot of the predicted cell-types from the PBMC data generated by each pipeline. Set size equals the total amount of predicted cell-types per pipeline. Intersection size representing the total amount of predicted cell-types that overlap between the intersected set of pipelines. The bottom right panel shows which pipelines are being intersected. **E** A barplot comparing the number of cells that are predicted to be a specific cell-type per pipeline

Next, we investigated the output of the pipelines using the large dataset of ~ 15,000 cells. The split-pipeline algorithm completed the analysis, however only 10 cells passed the QC thresholds and it was therefore not used in further analyses. After QC, the STARsolo, splitpipe and alevin-fry pipelines obtained just over 15,000 cells barcode of which 14,343 cell barcodes were identical between the pipelines (Fig. 4A). Additionally, when looking at gene and UMI content per cell, all pipelines perform similarly (Table 3, Fig S3A, B) especially STARsolo and splitpipe which give nearly identical results (Fig. 4B). Alevin-fry has a higher mean and median for both gene and UMI per cell content (Table 3), as well as more variance across the dataset (Fig S 3A, B, D). In order to further test the identity of the cells that are generated by these pipelines, we mapped the cells to a reference dataset using the R package Azimuth [31] and visualized the cells on a UMAP (Figs. 4C and 5A-C) showing distinct differences per pipeline. As the data consists of four donor samples, the data was split and integrated together on the donor level to remove as much bias by donor (Fig S3C). Subsequently, the cell type data generated by each pipeline was mapped using Azimuth [31] and visualized on an UMAP (Fig. 5A-C, Fig S3C). Across the three pipelines a total of 18 different cell types are predicted. Splitpipe has a total of 16 predicted cell types (Fig. 5B, D), STARsolo has a total of 15 predicted cell types(Fig. 5C, D), 14 of which overlap with those detected by splitpipe (Fig. 5D). Only the CD8 naïve and Plasmablast cell-types are missing from the STARsolo annotation (Fig. 5B, C, E). Conversely STARsolo has the proliferating NK cell-type annotated which is not annotated in the splitpipe data (Fig. 5B, C, E). Moreover, when looking at the number of cells per predicted cell type STARsolo and splitpipe are nearly identical sharing similar numbers cell counts for almost every predicted cell type (Fig. 5E). Alevin-fry splip however, shares only three predicted cell types (Fig. 5A, D). Moreover, most cell are predicted to be erythroid (Eryth) whilst this predicted cell type is not called in either splitpipe nor STARsolo datasets. The Azimuth algorithm provides cell type prediction scores and mapping scores, where cell type prediction score represents how well a cell maps to the closest cell in the reference data. The mapping score represent how well a cell is represented in the reference data a low score meaning that there are few cells that are identical within the reference map. When plotting the mapping and prediction scores (Fig S4) the alevin-fry data is performing poorly compared to the other pipelines, which made us conclude that alevin-fry splitp leads to low quality cell type assignment.

**Table 3:** Quantitative measurements per pipeline for the large dataset

Pipeline	Mean gene per cell	Median gene per cell	Mean umi per cell	Median umi per cell	N cells
alevin-fry	2785.3	2923	7297.8	6991.9	15620
splitpipe	2290.6	2283	6142.6	5493	15748
STARsolo	2176.1	2152	5689.2	5051	15875

To compare similarity, the data of alevin-fry, STARSolo and splitpipe pipelines were merged without integration and visualized (Fig. 4B). The result generated by alevin-fry splitp are completely separated by pipeline indicating that these results are very different. STARsolo and splitpipe followed the same pattern but still separate based by pipeline for most several cell types (Fig. 4B). The naïve B, and intermediate clusters of STARsolo and splitpipe mix well indicating highly similar results (Fig. 4B, C). This also occurs in the plasmacytoid Dendritic Cell (pDC) and Hematopoietic Stem and Progenitor Cell (HSPC) clusters and partially in the T regulatory (Treg), CD4 T Central Memory (TCM) and Mucosal-Associated Invarian T cell (MAIT) clusters (Fig. 4B, C). However, other clusters separate based on pipeline (Fig. 4B, C). To highlight the difference between the pipelines we performed a Pearson correlation of the expression data on the pseudo-bulk level (Fig S5). STARsolo and splitpipe correlate highly to each other, whereas alevin-fry differs greatly (Fig S5A). However, when we further performed a cell type cluster comparison between STARsolo and splitpipe; differences created by the pipelines were visible (Fig S5B). Additionally, differential expression was performed between the CD14 Monocytes and Natural Killer (NK) annotated cell types to investigate the differences. When analyzing the markers we noticed that several of the marker genes were either present in one but not in the other pipeline (Table S1, 2). By calculating the percent difference, we can see the magnitude of difference between two genes within the annotated cell types (Fig S6, Table S1, 2). Subsequently we compared the difference of total annotated features between pipelines (Table S3) showing that each pipeline aligns or counts the genes differently.

### Barcode extraction from synthetic data differs between algorithms

Two major steps are performed by the SPLiT-seq processing algorithms; alignment of transcriptomic reads and extraction of cell barcodes. For the purpose of comparing the ability of each algorithm to extract cell barcodes we created a synthetic dataset. This synthetic data contains a fixed number of cells (500) where each cell contains a specific number of cell barcode reads (10.000) ranging from perfect (harbouring no errors) to faulty (harbouring many errors). The CB correction distance is set in all pipelines to two or less bases. On the basis of this correction distance, reads are divided in two main categories; i) correctable reads, that contain two or less errors per barcode element, and ii) uncorrectable reads which contain three or more errors per barcode element. In this analysis, from the position-based algorithms STARsolo performs best and is able to capture more than half of the correctable linkers (Fig S7A). Splitpipeline and splitpipe perform worse and capture less than half of the correctable reads. zUMI’s and alevin-fry splitp performed worst with respect to barcode correction (Fig S7A). The linker based position and alignment algorithms LR-splitpipe and splitseq-demultiplex performed very well and enabled the capture of more than eighty percent of all correctable reads and the around twenty percent of the uncorrectable reads (Fig S7A). When comparing the sequence of the barcodes extracted LRsplitpipe, splitpipe, splitseqdemultipex and STARsolo find all the synthetic barcodes (Fig S7B). Interestingly splitpipe finds three additional CB and one different one. Alevin-fry performs badly only finding 249 barcodes. Splitpipeline finds 485 barcodes and similar to previous behavior all barcodes differ from other algorithms (Fig S7B).

## Discussion

**Fig. 6:**
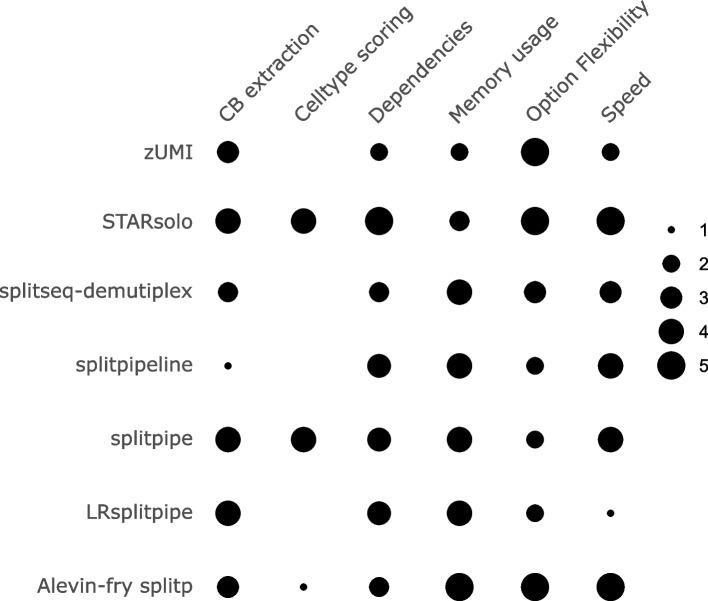
A qualitative comparison of functional features and results between pipelines. Graded from one to five. One representing worst and five best. CB extraction: A degree to which CB produced are similar to that produced by others. Dependencies: How many dependencies each pipeline has on other programs or packages that have the be installed before proper functioning. Memory Usage**:** Amount of RAM the program uses. Option Flexibility**:** The freedom users have to change settings of each run. Speed**:** How fast the pipeline finishes its analysis

SPLiT-seq is a relatively novel single cell sequencing technique, compared to droplet based single-cell sequencing, yet several pipelines have already been developed to process the data. Therefore, independent benchmarking and evaluation of these tools is of importance. In this study we compared a number of computational pipelines for the processing of SPLiT-seq data (Table 1) and graded them on several qualitative factors (Fig. 6). We do note however, that several factors of grading might be subject to experience bias and if repeated results might differ.

Due to the scalability of SPLiT-seq, the datasets that will be generated by this technique are expected to become larger and larger [1, 18, 35]. Therefore, runtime and memory performance should be evaluated. When speed of analysis is important STARsolo and alevin-fry perform best. However, as dataset size increases STARsolo’s RAM usage increases significantly, whereas alevin-fry can be used on very small amounts of RAM.

SPLiT-seq has the option of using different barcoding chemistries. When modifications to the protocol are performed STARsolo and zUMI provide complete freedom and customization (Table 1). The other pipelines have fixed settings for v1 or v2 chemistries. Another important feature is the option of collapsing of random hexamer reads to obtain correct cell barcode assignment. However, this feature is not built in every pipeline (Table 1), and a separate programming script is needed to perform this step, and is not a non-trivial task to implement. Depending on the programming language this can increase total runtime.

Several barcode extraction methods are used in the different pipelines and the results show that they all work similarly efficient except for the alignment method of splitseq-demultiplex [29], which generates much more barcodes than other pipelines (Table 2, Fig. 3A). Fixed position algorithms will always be computationally faster than alignment or linker-based position, as there is no additional alignment step that has to be performed. 

We found that in both the small mouse brain data as well as the PBMC data the amount of CB extracted between most pipelines are similar (Figs. 3A and 4A). However, differences in content of the cells was observed when visualizing data on a UMAP (Figs. 3B and 4B, C). Therefore, we suspect that the largest differences between pipelines are not produced by the barcode extraction methods but with the alignment and counting of the algorithms. Except for the alevin-fry splitp pipeline, all pipelines compared here use STAR software to align reads. STAR uses a Maximal Mappable Prefix approach [24], whereas alevin-fry uses k-mer based pseudo alignment [32]. This can cause significance downstream differences (Fig. 4B, C). Moreover, every pipeline uses different feature counting methods which might also contribute to differences output from the same data. Recently, more detailed comparisons have been made on efficiency of different aligners and feature counting tools with respect to single-cell data [33, 37–39], which is reflected in the outcome of the pipelines we compared here. Splitpipe and STARsolo have similar results in terms of cell type calling and differences that do occur are most likely due their different counting algorithms (Fig. 5, S5, 6). Whereas alevin-fry splitp, differing on both alignment and counting shows large differences and results in faulty cell type prediction (Fig. 5, S5, 6).

In addition to the mouse brain and human PBMC datasets, we compared these pipelines using a synthetic dataset which contains increasingly erroneous cell barcodes. We found that LR-splitpipe and splitseq-demultiplex performed best in correcting the CBs (Fig S7). However, when applied on larger datasets such as the mouse brain and human PBMC SPLIT-seq datasets, the LR-splitpipe and splitseq-demultiplex are found to be computationally intensive and cannot compete with STARsolo and splitpipe that are more efficient. Future implementation of these position based and alignment-based pipelines should focus on improving their speed and computational resource demand. Furthermore, we suggest a more thorough investigation on the possible types of errors, in addition to the ones we simulated in the synthetic dataset, that occur within CBs of real SPLiT-seq data. This will be instrumental in improving the existing pipelines, as well as the development of new ones.

At this point splitseq-demultiplex and splitpipeline are not well maintained and have to be updated to be able to handle newer barcode chemistries, thus we discourage the use of those pipelines [26, 29]. zUMI [25, 40] is more frequently maintained, however it has many dependencies on other packages that have been updated since the initial release of zUMI. Although zUMI [25, 40] provides a functional conda environment in which it runs, the packages and functionalities might be outdated.

When using the commercialized SPLiT-seq, we recommend using splitpipe as it is specifically designed to analyze SPLiT-seq data. Splitpipe contains several advantages over the other pipelines in terms of user friendliness. Several steps exist to confirm whether the correct options have been used reducing complexity for the user. In addition, several QC graphs can be produced on the user’s discretion. Whereas other pipelines offer more freedom with increased complexity. However, the splitpipe pipeline algorithm is only available after a purchase at Parse Biosciences. If unavailable we recommend using STARsolo [38] as it performs similarly to splitpipe, although random hexamer collapsing has to be performed separately. The difference most likely arises due to feature counting; however, STAR is a well-established alignment method and commonly used. Additionally, both STARsolo and splitpipe are computationally efficient in comparison with other pipelines and are therefore more scalable with the larger datasets SPLiT-seq experiments generate. If the SPLiT-seq library is sequenced using long read sequencing, we suggest using LR-splitpipe [27, 41], as it is specifically designed to handle long reads and copies several functions from the splitpipe pipeline.

SPLiT-seq offers an alternative to the droplet and plate based single-cell sequencing methods. It is also relatively easier, scalable and cheaper for large-scale experiments [35]. Despite these advantages, the development of analysis tools is still lagging behind and the existing tools are not benchmarked. Our comparative analysis of the current computational tools for SPLiT-seq data aids researchers to choose the most appropriate tool for data analysis.

## Methods

### Data retrieval

The small mouse brain dataset was retrieved from NCBI [GSM3017260] [18] containing one sub-library with 77,621,181 reads. The large PBMC dataset was published [35] and sequencing data available on request from parse biosciences. The large PBMC consists of two sub-libraries of which only the second one, containing 1,704,418,175 reads, was used. The quality of both datasets was first analyzed with fastQC and did not require any trimming.

### Pipeline comparison

All pipelines except zUMI, were run on the Snellius SURF Dutch research cluster. SLURM was used to run batch jobs, limit memory usage and measure computational performance. Memory limits were set to 64 Gb and 256 Gb for the small and large dataset respectively with one exception for STARsolo, which was allowed up to 1TB of RAM to allow completion. Each pipeline was run a total of 5 times to obtain performance averages.

All pipelines were set to perform as similarly as possible; cell barcode correction distance was set to two and only kept cells barcodes that have more than 100 reads assigned to them.

As each pipeline performs the analysis steps in a certain order a short description is given about each pipeline is provided below and a graphical depiction is displayed in Figure S1. To simplify the steps names are reduced to core function of the steps.

### STARsolo

STARsolo (2.7.10a) [24, 38, 42] was run using the CB_UMI_Complex option, which allows for position-based input of the CB and UMI. Index position for the BC 3 and 2 were 10 to 17 and 48 to 55 respectively. For v1 chemistry BC 1 position was 86 to 93 and for v2 chemistry 78 to 85. UMI position was set to index 0 to 9. CBs were corrected to a position-based whitelist with the CB correction option of Edit_dist_2 which sets the correction distance to two base pairs.

STARsolo runs its analysis steps in the following order: Alignment of reads to genome, CB extraction and correction, Counting UMIs, counting features, create single cell matrices (Fig S1).

### Splitpipeline

All the splitpipeline (0.0.1) [18, 26] base options were used except for the chemistry used, which was set to the respective chemistry of the input data with the –chemistry option. Cell barcode edit distance is hard coded to two base pairs.

Splitpipeline runs its analysis steps in the following order; CB extraction and correction, Collapsing of CB, Counting UMIs, Alignment of reads to genome, counting features, create single cell matrices (Fig S1).

### Splitpipe

Splitpipe (v1.0.3p) [35] performs an internal check of the chemistry and kit used by taking a subsample of the dataset, which provided the same information that was given upon data request (kit WT, chemistry v2). Splitpipe automatically tries to use an Anaconda virtual environment, which was turned off using the –start_timeout 0 option. Otherwise, no additional options were used. Cell barcode edit distance is hard coded to two base pairs.

Splitpipe runs its analysis steps in the following order; CB extraction and correction, Collapsing of CB, Counting UMIs, Alignment of reads to genome, counting features, create single cell matrices (Fig S1).

### zUMI

A zUMI (2.9.7d) [25, 40] yaml file was generated using the Rshiny app provided on their github. CB position was set to read 2 with indices 11–18, 49–56 for BC3 and BC2, respectively. For BC1 with the indices 79–86 and 87–94 were used for v2 and v1 chemistries respectively. UMI positions indices were set to 1–10. Cell barcode edit distance was set to two and were corrected to a provided cell barcode whitelist. We were unable to make zUMI working on the Snellius cluster due to dependency conflicts, therefore we utilized a smaller local server where we could use the internally provided Anaconda virtual environment (Fig S1).

zUMI runs its analysis steps in the following order; CB extraction and correction, Counting UMIs, Collapsing of CB, Alignment of reads to genome, counting features, create single cell matrices.

### SCSit

SCSit [28, 43] test runs were made using the steps provided on their github with both their test data and other data. All runs crashed with a segmentation fault error. Troubleshooting was performed but no obvious cause was found to be the source, after which authors were notified Fig S1).

### Splitseq-demultiplex

The run type (-v) of Splitseq-demultiplex (0.2.1) [29] was set to merged which performs the BC Alignment type CB extraction. Random hexamer collapsing (-c) was set to true and CB edit distance(-e) to two base pairs. Depending on the chemistry used barcode sequences were changed by providing premade input files (-1, -2,-3).

SPLiTseq-demultiplex runs its analysis steps in the following order; CB extraction and correction, Collapsing of CB, Counting UMIs, Alignment of reads to genome, counting features, create single cell matrices (Fig S1).

### Alevin-fry splitp

First random hexamer collapsing was performed using splitp (0.1.0), for v1 chemistry positions 78 to 94 and for v2 chemistry positions 79 to 86 were used. Subsequently alignment and CB correction were performed with salmon (1.9.0) and alevin-fry according to the alevin-fry SPLiT-seq tutorial. Alignment was set to –sketch with the –tgMap to map to gene features. Subsequently Alevin-fry (0.8.1) generate-permit-list was used to find all barcodes within a given whitelist. Alevin-fry collate to count UMIs and subsequently Alevin-fry quant to count features and generate a matrix [44] (https://github.com/COMBINE-lab/splitp, https://github.com/COMBINE-lab/alevin-fry, https://github.com/COMBINE-lab/salmon).

Alevin-fry splitp runs its analysis steps in the following order; Collapsing of CB, Alignment of reads to genome and extraction of CB, CB correction, counting features, create single cell matrix (Fig S1).

### LR-splitpipe

The LR-splitpipe [27, 41] pipeline was slightly modified to turn of negative strand alignment. In addition, v1 chemistry options were added. Otherwise, all base options were used with a hard coded CB edit distance of two. Output of LR-splitpipe is a fastq file with the corrected CB sequence which was subsequently used for alignment with STARsolo to generate a single cell matrix.

LR-splitpipe runs its analysis steps in the following order. Extraction of CB and UMI, correction of CB, generate corrected fastq. After which it follows order of STARsolo (Fig S1).

### Random hexamer collapsing

Not all pipelines perform random hexamer collapsing. To address random hexamer collapsing for pipelines that did not perform this step, a custom R script was written [10.5281/zenodo.8362859]. In the first round a total of 96 barcodes are used. The first 48 are assigned to polyA capturing oligos whereas the last 48 are assigned to random hexamer capturing oligos. The 1st, index 1, polyA barcode is linked to the 1st, index 49, random hexamer barcode, and repeated for each following barcode combination. The Rscript numerates the barcode sequences to simplify and speed up computation. Collapsing occurs by subtracting 48 from each number that is larger than 48, after which the numerated BC1, 2 and 3 numbers were pasted together. Subsequently the list of barcode sequences was collapsed by looking for duplicate barcodes and performing row sums of the respective data matrix indices and stored into a new matrix with collapsed cells.

### Quality control

Seurat [31] was used to analyze each dataset. For the small dataset a minimum of 1000 and maximum of 10,000 features were used as a threshold for each cell. Additionally, a maximum of 5% mitochondrial reads was allowed. For the large dataset a minimum of 600 and maximum of 5000 features was used as a threshold of each cell. Subsequently data was merged or kept separately and treated as thus; Data was normalized using log Normalization and a scale factor of 10,000. A total of 2000 variable features were found using variance stabilizing transformation (vst) after which data was scaled. PCA was performed and subsequently nearest neighbours and UMAP were calculated with the first 10 and 40 PCAs in the small and large dataset respectively. For the large dataset batch correction was performed per donor sample using reciprocal PCA [31] and cell types were called using the Azimuth [31] package. Subsequently Leiden clustering was used to find the most frequent cell type annotation in each cluster, if the most frequent assignment within that cluster was not more than 25% of the total cluster the cell type annotation was removed. Visualization was performed using UpSet and ggplot2.

### Gene–gene correlation

Pseudobulk data per pipeline or cell-type group was generated using the AggregateExpression function in the Seurat R (v5.0) package. Expression was subsequently tested against each other using the base correlation function (cor) in R using the Pearson method. Result was visualized using a heatmap.

### Synthetic data generation

To generate the synthetic SPLiT-seq data we concatenated the six specific elements that are present in the SPLiT-seq cell barcode sequence. These are in following order, UMI, BC3, LINKER2, BC2, LINKER1, BC1. Each cell barcode that was synthesized was given 10.000 amount of reads. Each read was given a UMI that had a hamming distance greater than two for every other UMI within a synthetic cell. This was used to prevent UMI collapsing of reads performed by some pipelines. All reads within a synthetic cell were divided equally into several categories (read type), where each category was given a specific 120 base sequence of a known gene so that the retrieval of a category could be measured in the count matrix on a per feature basis (Table S4). A total of eight categories were created; i) Perfect reads that do not contain any errors. ii), iii) and iv) reads that contain correctable barcode elements in one, two or all three barcode element positions. v), vi) and vii) reads that contain uncorrectable barcode elements in one, two or all three barcode element positions and viii) a completely random sequence. As the Levenshtein correction distance in each pipeline is set to two we divided reads in two major categories; i) correctable barcodes that contain two or less substitution errors and should be within correction distance and ii) uncorrectable barcodes that contain three or more substitution errors and are uncorrectable. Errors were introduced by random substitutions of bases in a single or multiple barcode sequence elements. All reads were written to a fastq file format with all easily retainable information such as read category, or original barcode were stored in the read name.

### Supplementary Information

**Supplementary Material 1.**

**Supplementary Material 2.**

**Supplementary Material 3.**

**Supplementary Material 4.**

**Supplementary Material 5.**

## Data Availability

The small mouse brain dataset was retrieved from NCBI [GSM3017260], the large human PBMC dataset was received upon request from Parse Biosciences.
