# Supplementary material for: Split Pool Ligation-based Single-cell Transcriptome sequencing (SPLiT-seq) data processing pipeline comparison
Source: BMC Genomics. 2024 Apr 12;25:361. doi: 10.1186/s12864-024-10285-3 (PMC11010347; doi:10.1186/s12864-024-10285-3)
Supplement: Supplementary file 1 — Supplementary Material 1. [file 12864_2024_10285_MOESM1_ESM.docx]

**Supplementary Figures**


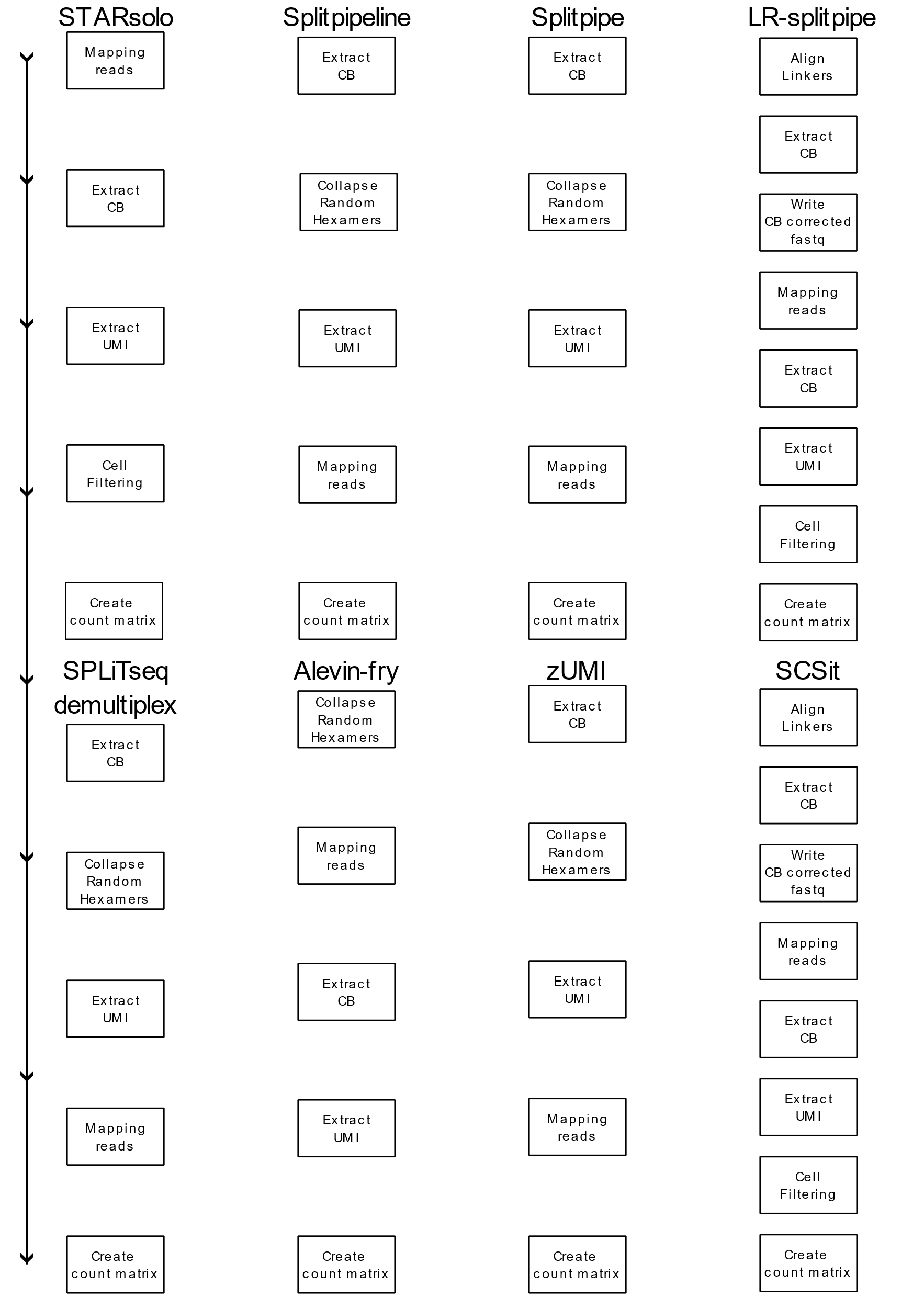


**Supplementary Figure 1**: Order of steps per pipeline. General features are used instead of coding function to make it more readable.


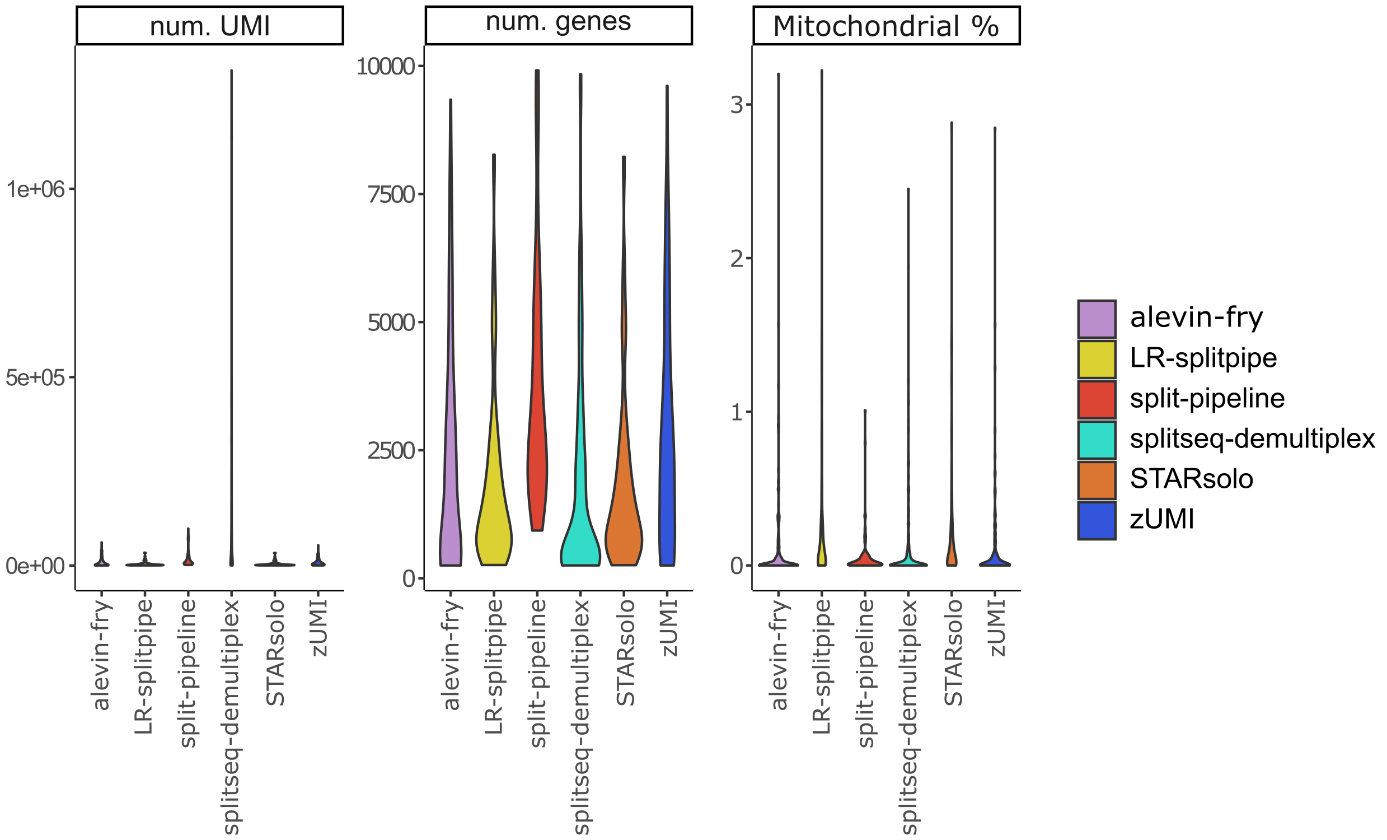


**Supplementary Figure 2:** QC measurements of the small dataset. Number of UMI, Gene and percent mitochondrial read per cell.


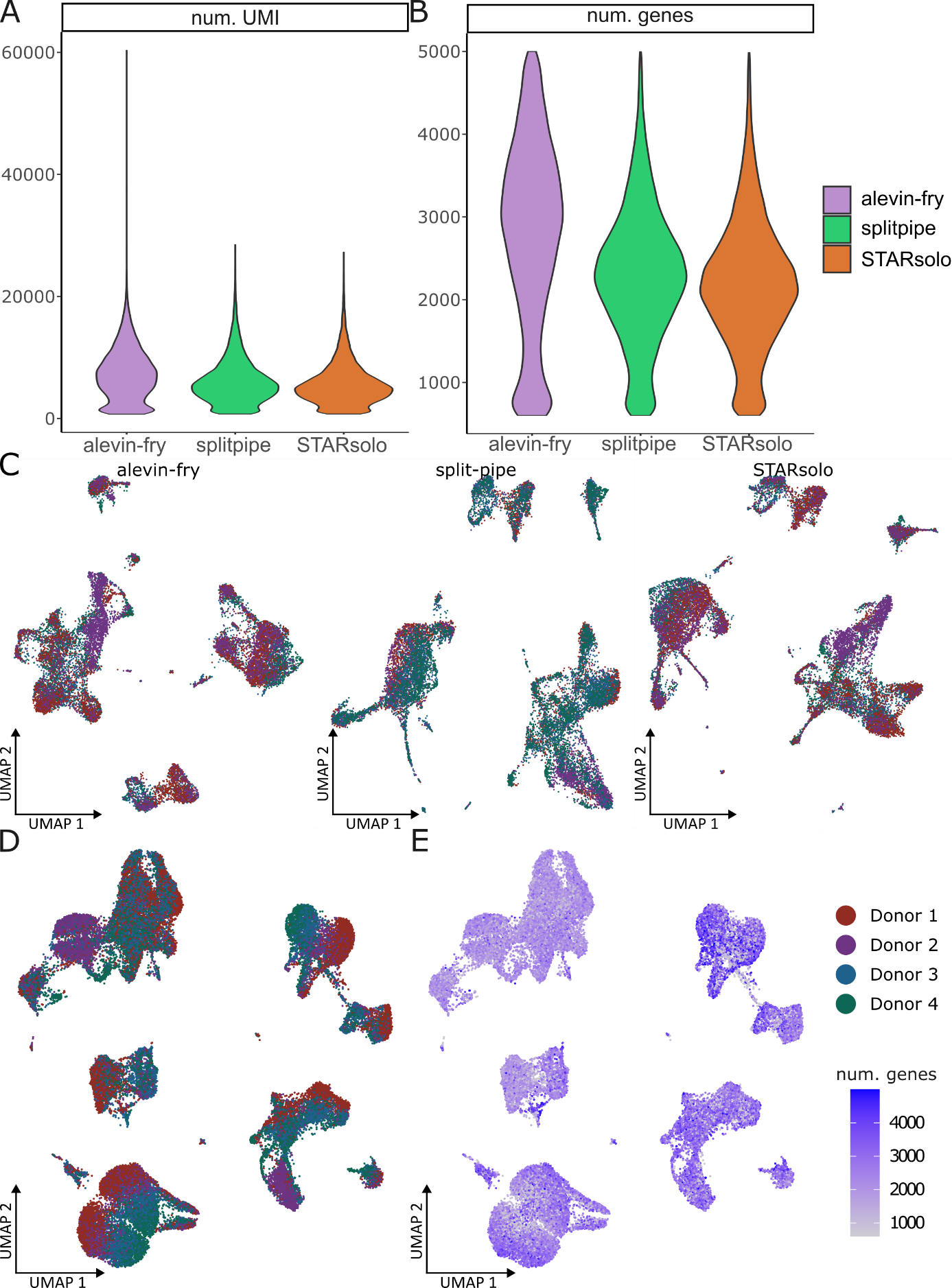


**Supplementary Figure 3:** QC measurements of the large dataset. **A.** Number of UMI per cell. **B.** Number of gene per cell. **C.** UMAP of the data generated by each pipeline separately, colored by donor sample. **D.** UMAP of the data generated by each pipeline merged together colored by donor sample. **E.** Number of gene per cell.


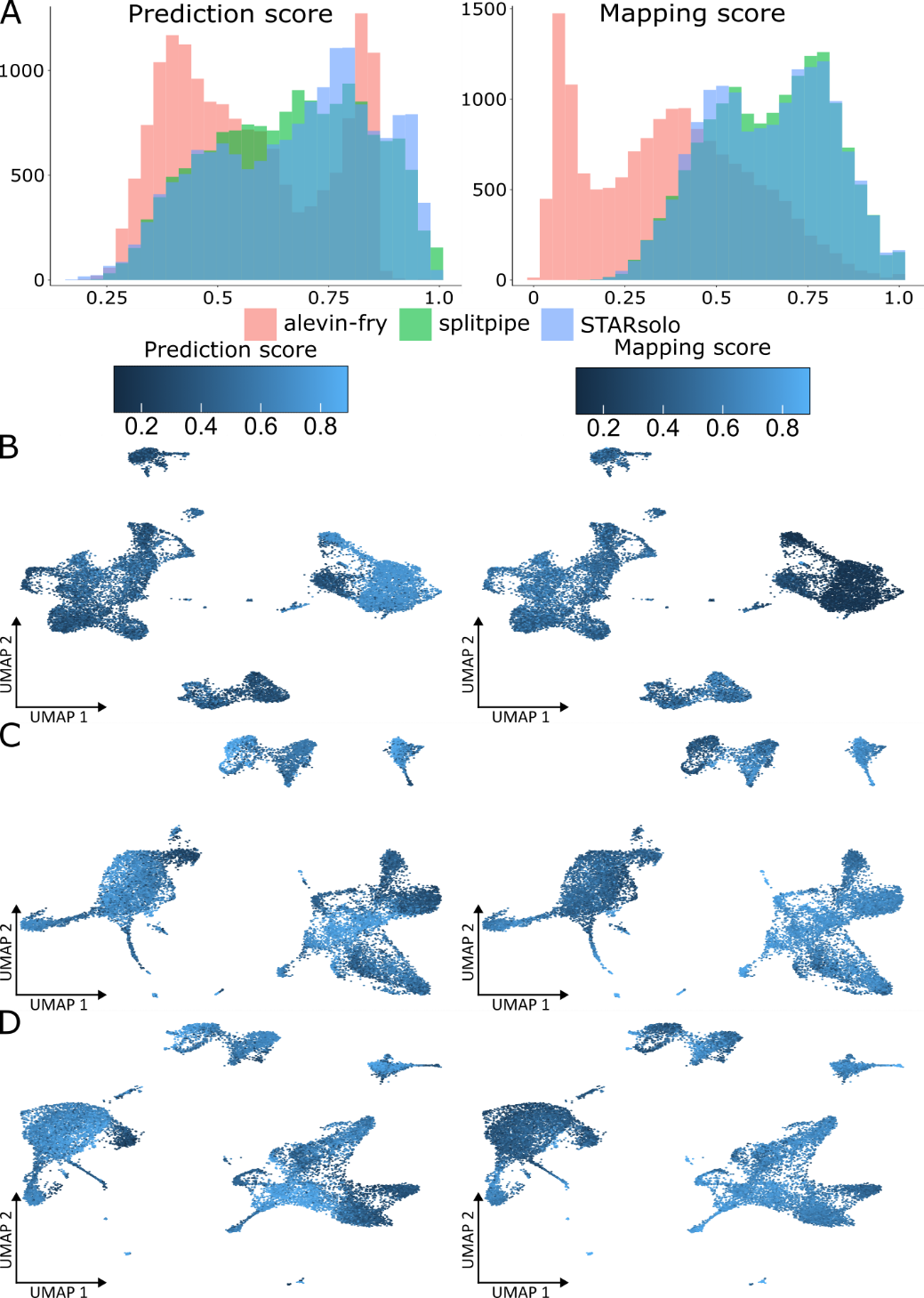


**Supplementary Figure 4:** **A.** Histograms of the level 2 prediction scores and mapping scores generated by the Azimuth R annotation package. Colored by pipeline. **B.** UMAP of each pipeline separately colored by *left* level 2 prediction score and *right* mapping score. **B.** alevin-fry splitp**. C.** splitpipe **D.** STARsolo.


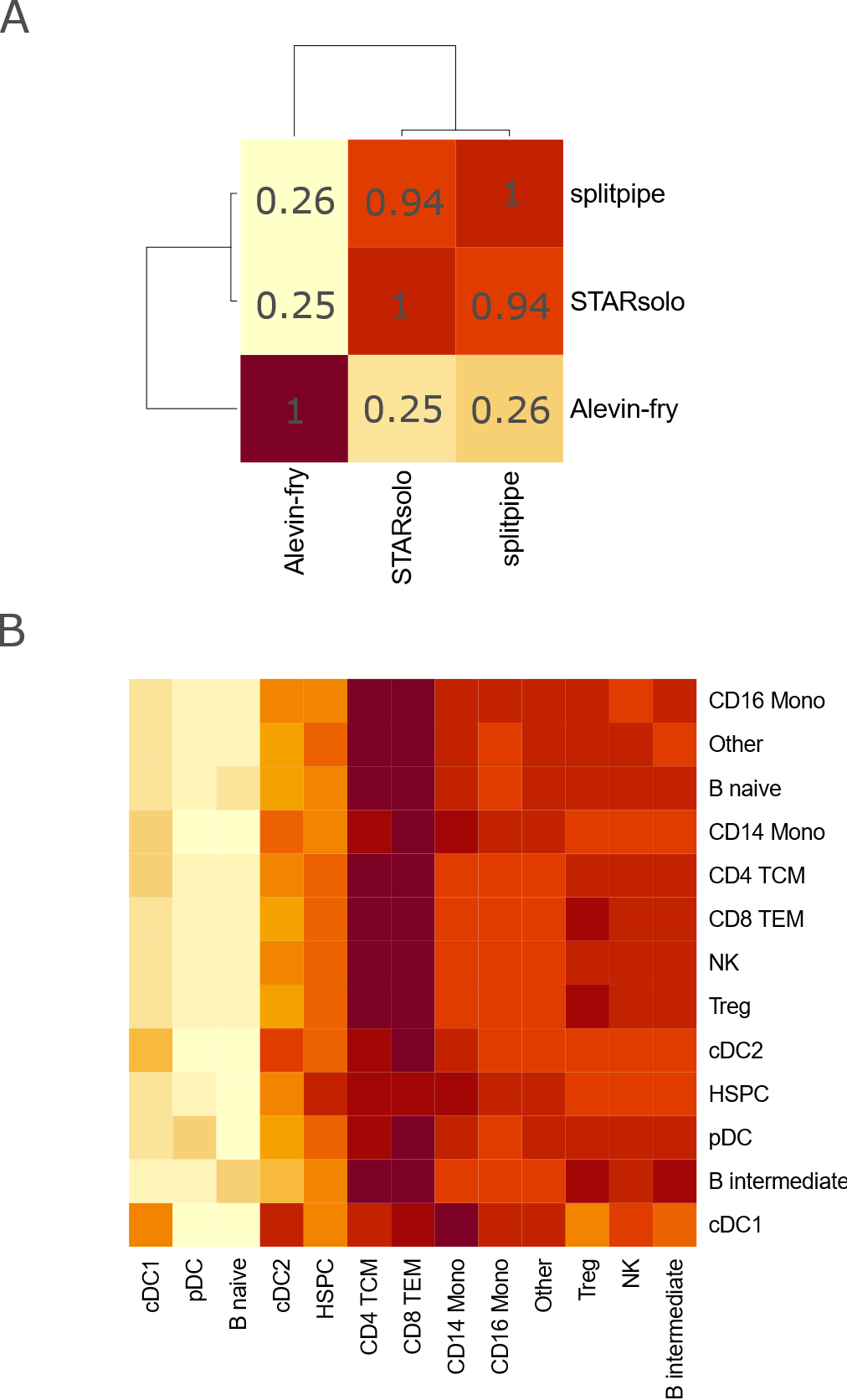


**Supplementary Figure 5. A.** Pearson correlation based on gene expression values extracted from the pseudobulk of the single cell data created by each pipeline. **B.** Pearson correlation of gene expression on the pseudobulk level of for each celltype cluster between the STARsolo and splitpipe pipelines.


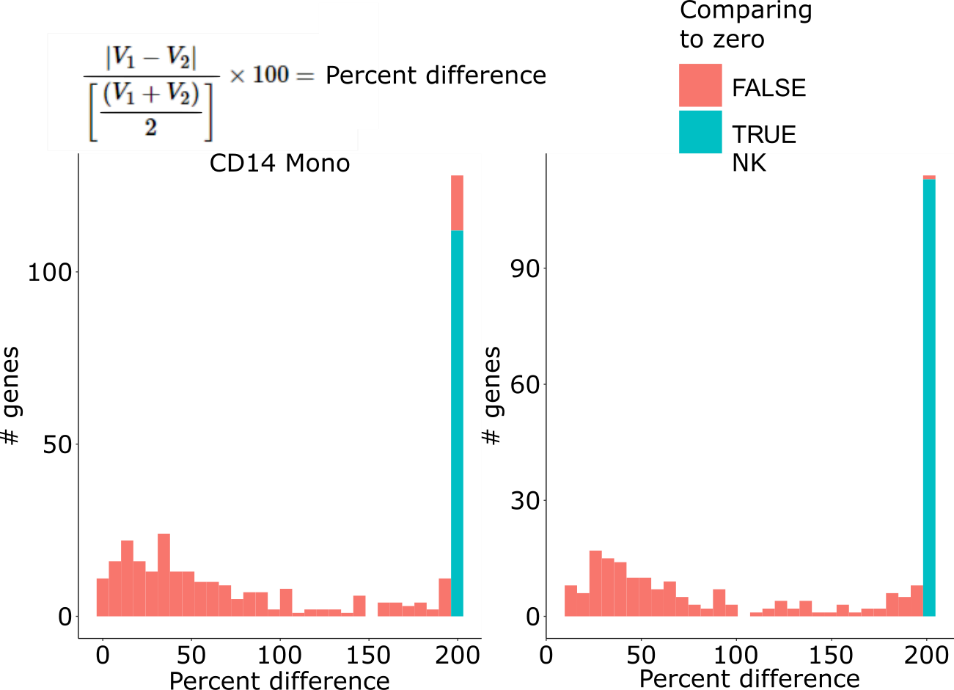


**Supplementary Figure 6.** Histograms of the percent difference of marker genes between STARsolo and splitpipe within a celltype group. *Left* CD14 Monocytes. *Right* Natural Killer cells. Histograms are colored to show genes that contain a zero in the calculation. Formula to calculate the percent difference is provide at the top left.

**Supplementary Table 1.** The Percent Difference of marker gene expression between STARsolo and splitpipe processed cells of the CD14 Monocytes assigned cluster.

**Supplementary Table 2.** The Percent Difference of marker gene expression between STARsolo and splitpipe processed cells of the Natural Killer cell assigned cluster.

**Supplementary Table 3.** Total difference in detected features per pipeline for the large PBMC dataset.


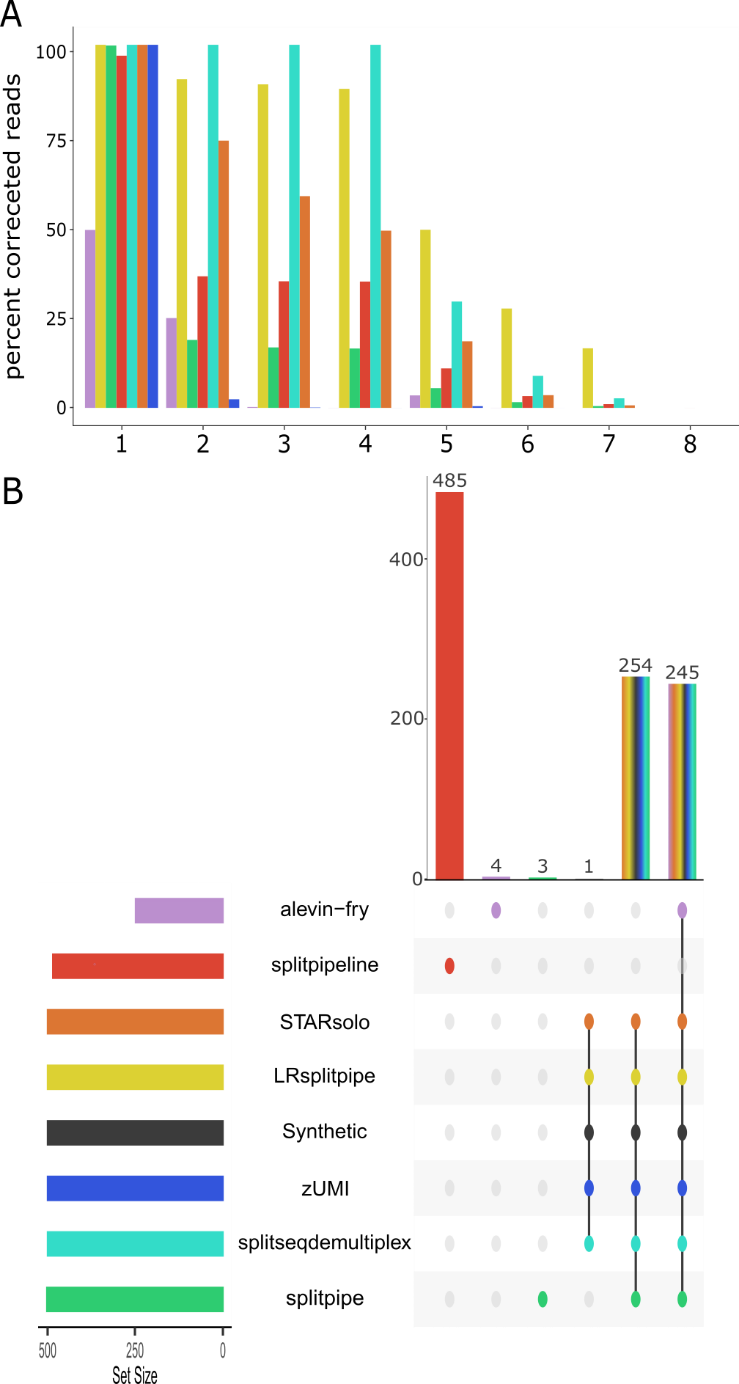


**Supplementary Figure 7. A.** Percent captured synthetic reads for 8 read categories. 1. Perfect reads. 2. Two or less substitutions in one barcode region. 3. Two or less substitutions in two barcode region. 4. Two or less substitutions in three barcode regions. 5 More than two substitutions in one barcode region. 6. More than two substitutions in two barcode regions. 7. More than two substitutions in three barcode regions. 8. Random sequence of DNA **B.** Upset plot showing the intersect of the captured CBs by each pipeline of the synthetic data.

**Supplementary Table 4.** Genes and their sequences used as read type annotation for the synthetic dataset.
